# Augmented and virtual reality in dental and oral and maxillofacial surgery education: a systematic review with a taxonomy of training technologies

**DOI:** 10.1186/s12909-025-08561-1

**Published:** 2026-02-23

**Authors:** Mathias Maes, Jasper Deferme Van Gerven, Robin Willaert, Reinhilde Jacobs

**Affiliations:** 1 Faculty of Medicine, University of Leuven, Leuven, Belgium; 2 OMFS IMPATH Research Group, Department of Imaging and Pathology, Faculty of Medicine, KU Leuven, Leuven, Belgium; 3 Department of Oral and Maxillofacial Surgery, University Hospitals Leuven, Leuven, Belgium; 4 Department of Dental Medicine, Karolinska Institute, Stockholm, Sweden

**Keywords:** Augmented reality, Virtual reality, Surgical training, Simulation-based education, Systematic review

## Abstract

The use of augmented reality (AR) and virtual reality (VR) in surgical training has increased significantly. Trainees in skill-intensive specialties such as dentistry and oral and maxillofacial surgery (OMFS) highly depend on treating real life cases under supervision. This old apprentice model is challenged by reduced learning opportunities and patient safety questions. AR and VR training can create alternative learning opportunities and intricate skill acquisition by simulating scenarios, providing real-time feedback and repetitive practice without patient risk. This systematic review aims to evaluate the effectiveness and usability of AR/VR tools in surgical skills training and provides a clear overview of the available systems. In accordance with the PRISMA-SR guidelines, different databases were searched for studies published between April 2015 and 2025. Different AR and VR tools were assessed from twenty-five RCTs and categorized into three groups: haptic simulators, head-mounted devices, and software platforms. Bias assessment via the RoB-2 tool for randomized trials revealed an overall body of evidence with a low risk of bias. Most studies have shown equal or superior outcomes of AR/VR versus conventional training, particularly in terms of preparation accuracy, diagnostic precision, and learner confidence. A minority reported comparable results without clear advantages. In conclusion, AR/VR technologies show promise for enhancing surgical training, although methodological heterogeneity limits comparability. Further research with long-term RCTs, standardized outcomes and user satisfaction is therefore necessary.

**Graphical Abstract:**

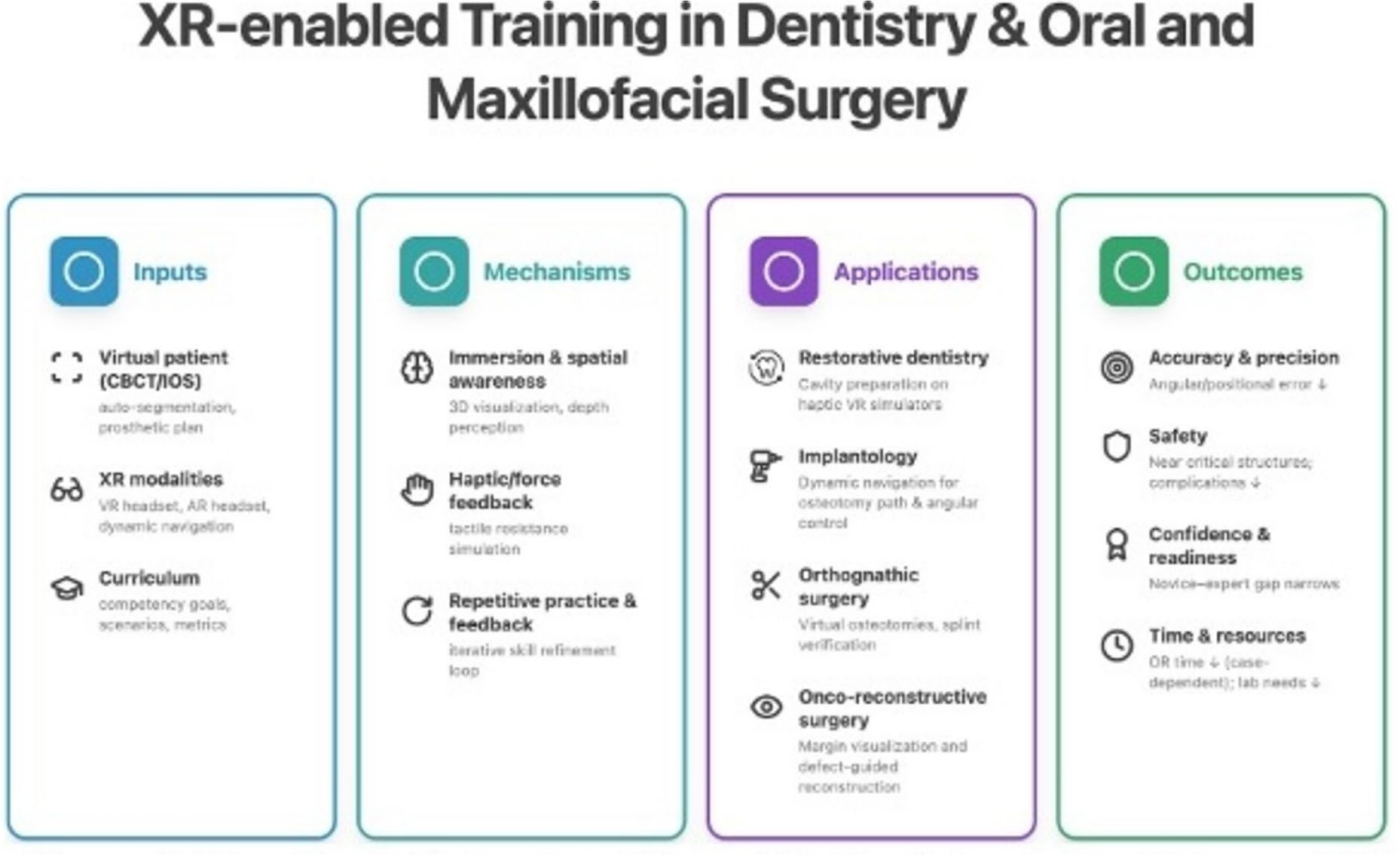

**Supplementary Information:**

The online version contains supplementary material available at 10.1186/s12909-025-08561-1.

## Introduction

Virtual reality (VR) and augmented reality (AR) are increasingly used in surgical education. They provide immersive environments for knowledge acquisition and skill development. VR places the learner in a fully computer-generated environment, typically via a head-mounted display. AR overlays digital information onto the real world, often via smart glasses, smartphones, or tablets. Haptic interfaces (e.g., gloves or controllers) can be used in both VR and AR to deliver tactile feedback through vibration, force, or resistance and to improve interaction with virtual objects.

Dentistry and oral and maxillofacial surgery (OMFS) training require complex knowledge and fine motor skills within limited timeframes, which is challenging for novice learners [1]. Training traditionally relies on supervised practice with real patients. This model is increasingly constrained by workload, time pressure, and patient-safety considerations, which reduce hands-on learning opportunities [2–4]. VR and AR tools are currently being used to effectively replicate operating room scenarios, provide real-time feedback, and allow repetitive practice without patient risk [5]. Applications already span preoperative planning, implantology training, and restorative dentistry [6–8].

Within OMFS, AR has been reported since 1995 and is now applied across several domains. Orthognathic surgery and dental implantology remain prominent areas, with growing use in head and neck oncologic resection and reconstruction. AR-based teaching tools also support visualization of complex anatomy [9]. In head and neck oncology, AR navigation can overlay tumor margins and critical structures intraoperatively [10]. In implantology, dynamic navigation has been associated with improved placement accuracy compared with freehand techniques, supporting novice performance [11]. Mixed-reality systems in orthognathic contexts have been linked to improved planning accuracy, reduced intraoperative time, and enhanced trainee knowledge and technical skills [12].

Extended reality (XR) technologies have been associated with improved procedural accuracy and efficiency in surgical education [13–15]. Beyond performance metrics, VR-based learning has been linked to higher motivation, enjoyment, confidence, and post-intervention knowledge gains [16–19]. High-fidelity XR platforms may enable simulation of rare procedures, support robotic-assisted training, and facilitate remote mentorship [20, 21]. Cost-effectiveness has been suggested, with lower per-trainee costs than cadaveric or animal-laboratory sessions, although initial investment can be substantial [22–24].

Despite increasing implementation, evidence on whether AR and VR training improves objective technical performance compared with conventional methods in dental and OMFS education remains fragmented [25, 26].

This systematic review aimed to determine whether AR- and VR-based training improves objective educational performance compared with conventional teaching methods in dental and OMFS education. Given the heterogeneity of training tasks and assessment methods, the primary outcome was defined as the main performance-based educational endpoint prespecified by each randomized controlled trial, typically reflecting objectively assessed technical skill, procedural accuracy, error rates, or task-specific competence, as summarized in Table 2. Secondary aims were to evaluate effects on learner-related outcomes, including knowledge acquisition and retention, self-efficacy, confidence, cognitive load, and user satisfaction, and to examine differences in educational effectiveness across AR/VR tool categories.

## Methods

### Protocol and registration

This systematic review was conducted in accordance with the Preferred Reporting Items for Systematic Reviews and Meta-Analyses (PRISMA) guidelines [27] and was registered on the Open Science Framework (OSF). To enhance transparency, the study protocol was prospectively uploaded to OSF (10.17605/OSF.IO/CXKSA).

### Eligibility criteria

- Population (P): Undergraduate dental students, postgraduate residents and fellows in dentistry and OMFS.
- Intervention (I): AR or VR-based training tools.
- Comparator (C): Traditional training (didactic, cadaver labs, non-AR/VR simulators).
- Outcomes (O):
  - Primary outcomes were objective, performance-based measures of surgical skill (e.g., accuracy, error rates, procedural quality or time).
  - Secondary outcomes included knowledge retention and learner-reported measures such as confidence or satisfaction.

## Study design

Randomized controlled trials, including parallel-group, cluster-randomized, and crossover designs, published in English between January 2015 and April 2025 were included. We restricted inclusion to RCTs to maximize internal validity when comparing AR/VR training with conventional methods.

## Exclusion criteria

Non-randomized study designs, including quasi-randomized trials or controlled trials without true random allocation, were excluded; pre–post studies without a parallel control group were excluded by design; non-educational applications of AR/VR; studies not involving dental or OMFS learners; interventions without an AR/VR/XR component; studies in which no objective, performance-based primary outcome was assessed; studies evaluating only secondary outcomes such as learner confidence, perceptions, or satisfaction without an objectively measurable clinical skill outcome; secondary literature (reviews, editorials); conference abstracts or records without full text; non-human studies; and publications outside the predefined language and date limits.

## Databases and search criteria

A systematic search was conducted in PubMed, the Cochrane Library, and Web of Science on 15 June 2025. The search combined controlled vocabulary terms (MeSH, where applicable) and free-text keywords related to immersive technologies, education, and the clinical domain. Boolean operators were consistently applied, using OR to combine synonyms within the same concept and AND to link different conceptual domains. The key terms were combined as follows: (‘virtual reality’ OR ‘augmented reality’ OR ‘mixed reality’ OR ‘extended reality’ OR ‘VR’ OR ‘AR’ OR ‘XR’) AND (‘dental education’ OR ‘dentistry’ OR ‘education’ OR ‘training’ OR ‘simulation’) AND (‘oral surgery’ OR ‘oral and maxillofacial surgery’). Search strategies were tailored to the indexing system of each database. The complete search strategies for each database are presented in Table 1.

**Table 1:** Overview of the search strategies per database

Database	Search Terms Used	Records Identified
Cochrane Library	(“Virtual Reality” OR “Augmented Reality”) AND (“Education” OR "Training") AND (“Surgery, Oral” OR “Dentistry” OR "OMFS" OR "Maxillofacial")	29
PubMed	("Virtual Reality"[MeSH] OR "Augmented Reality"[MeSH] OR "Simulation Training"[Mesh] OR “VR” OR “AR” OR "virtual reality" OR "augmented reality" OR “Simulation” OR “Simulator”) AND (("Education, Dental"[MeSH] OR "Surgery, Oral/education"[MeSH] OR "dental education" OR "oral surgery education" OR "maxillofacial surgery education") OR ((“Education”[MeSH] OR “education” OR “training”) AND (“Dentistry”[MeSH] OR “Surgery, Oral”[MeSH] OR “dentistry” OR “OMFS” OR “maxillofacial” OR “oral surgery”)))	116
Web of Science	TS = (("Virtual Reality" OR "Augmented Reality") AND ("Education" OR "Training") AND ("Oral Surgery" OR "Dentistry" OR "Maxillofacial" OR "OMFS"))	245

Searches were limited to English-language publications published between January 2015 and June 2025, with results restricted to randomized controlled trials using database filters. Grey literature sources, including conference proceedings, theses, and preprints, were not searched. Reference lists of all included full-text articles were manually screened; however, no additional randomized controlled trials meeting the predefined eligibility criteria were identified.

## Data collection process

**Fig. 1:**
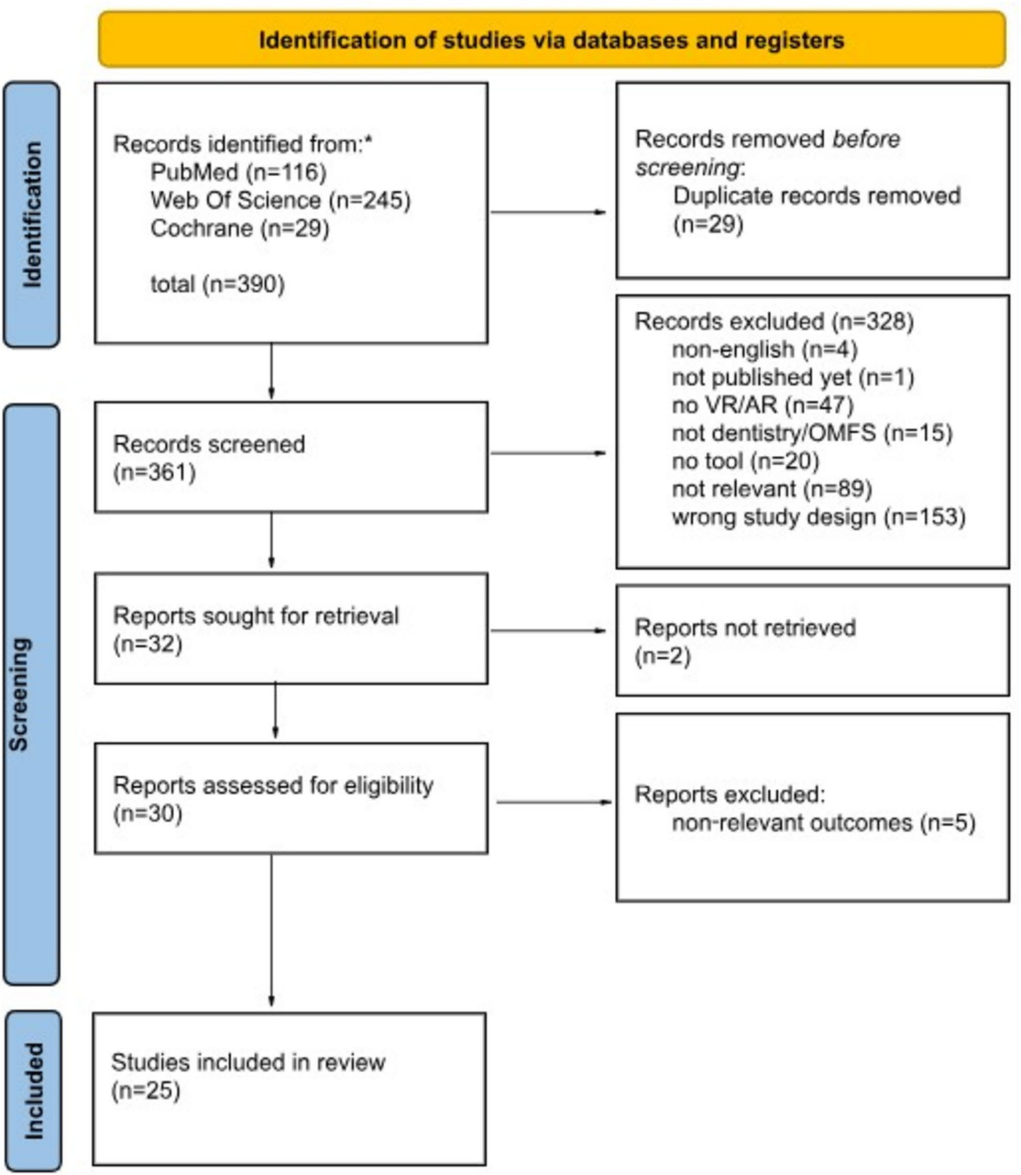
PRISMA flowchart

Study selection and screening procedures are summarized in the PRISMA flow diagram (Fig. 1). Two reviewers independently screened titles and abstracts for eligibility. Inter-reviewer agreement was high (Cohen’s κ = 0.86). Discrepancies were resolved through discussion until consensus was reached.

## Data extraction and taxonomy

Data extraction was performed independently by two reviewers using a standardized extraction form. The extraction form was pilot tested on a subset of included studies and refined accordingly prior to full data extraction. Discrepancies were resolved through discussion until consensus was reached.

For each included study, core study characteristics and outcomes were extracted (Table 2). In addition, details of the AR/VR training technologies were systematically classified to establish a taxonomy of educational interventions evaluated in the included trials. Extracted variables included device type (e.g., head-mounted displays, haptic simulators, software-based platforms), commercial availability, hardware and software components, fidelity characteristics (visual, haptic, and/or auditory), and the targeted training scenario. This taxonomy is presented in Tables 3, 4 and 5.

**Table 2:** Characteristics of the included studies according to tool performance

Study (Author, Year)	Simulator (Type)	Design	Population	Primary Outcome	Secondary Outcome	Primary result
Daud A et al., 2023 [28]	VRHS (haptic VR)	2-arm crossover	23 Year-3 dental students	Quality of Class I cavity preparations (VRHS haptic simulator vs phantom-head training)	Learner satisfaction and perceived realism	No statistically significant difference
de Boer IR et al., 2017 [29]	Simodont (haptic VR)	Crossover RCT	101 dental students	Cavity preparation performance and learner satisfaction (Simodont haptic VR simulator with force-feedback vs no force-feedback)	Student satisfaction and perceived usefulness	Favours AR/VR
Dwisaptarini AP et al., 2018	Visuo-tactile VR task	2-arm RCT	32 Year-6 dental students	Minimally invasive caries removal accuracy (visuo-tactile VR simulation vs conventional model)	Not reported	No statistically significant difference
Fu J et al., 2024 [30]	UniDental (haptic VR)	3-arm RCT	108 dental students	Residual calculus amount and overall scaling performance score (ultrasonic scaling training, haptic VR)	Student perceptions of realism, usability, and learning effectiveness	Favours AR/VR
Hamama H et al., 2024 [31]	Haptic VR cariology simulator	2-arm crossover RCT	76 dental students	Caries removal knowledge and lesion detection accuracy (haptic VR cariology training)	Self-confidence, perceived learning benefit	No statistically significant difference
Hu J et al., 2025 [32]	REP-VS (interactive VR)	2-arm RCT	123 Year-4 dental students	Regenerative endodontics theory exam and practical procedure scores (interactive VR pulp regeneration simulation)	Learning motivation, satisfaction	Favours AR/VR
Huang S et al., 2023	ChatGPT-assisted VR	2-arm RCT	187 dental students	Clinical procedure performance score (ChatGPT-assisted immersive VR)	Motivation, self-efficacy, cognitive load	Favours AR/VR
Im JE et al., 2024	3D VR (Oculus Quest 2)	2-arm RCT	79 dental hygiene students	User-experience rating (immersion/usability for 3D intraoral radiography VR vs 360° video)	Usability, satisfaction	Favours AR/VR
Li Y et al., 2025 [33]	VR simulators (Unidental & Simodont)	Hybrid RCT	≈30 Year-4 dental students	Veneer preparation quality (Unidental/Simodont VR vs phantom-head training)	Perceived realism, educational value, satisfaction	No statistically significant difference
Lu J et al., 2023	Virtual simulation platform (pulpotomy)	2-arm RCT	199 Year-4 dental students	Pulpotomy theoretical exam and operative skill score (virtual simulation experimental platform)	Not reported	Favours AR/VR
Mansoory MS et al., 2022 [34]	3D VR (Gear VR)	2-arm RCT	50 dental students	Neutral-zone design knowledge test score and learner satisfaction (Gear VR mixed reality)	Learner perceptions and confidence	Favours AR/VR
Mladenovic et al., 2019 [35]	Mobile augmented reality (AR) simulator (smartphone app)	Randomized controlled trial	41 Year-4 and 5 dental students	Inferior alveolar nerve block knowledge/skill (post-training questionnaire)	Procedure time and anesthesia success rate	Favours AR/VR
Murbay S et al., 2020 [36]	Simodont (haptic VR)	2-arm RCT	32 Year-2 dental students	Class I/II cavity preparation accuracy (Simodont haptic VR vs phantom-head training)	Not reported	Favours AR/VR
Peters P et al., 2024	HMD-based immersive VR	3-arm RCT	101 novice students	Surgical suturing OSATS score and self-assessed competence (HMD VR simulation vs e-learning)	Cognitive load, confidence	No statistically significant difference
Philip N et al., 2023 [37]	SIMtoCARE Dente (haptic VR)	2-arm RCT	14 Year-4 dental students	Pulpotomy access outline and chamber deroofing quality (SIMtoCARE Dente haptic VR vs conventional)	Student perceptions, satisfaction, confidence	No statistically significant difference
San Diego JP et al., 2022 [38]	Haptic VR (visuo-tactile)	Cluster RCT	64 Year-1 dental students	Cavity preparation quality and instrument handling (Simodont haptic VR vs phantom-head)	Not reported	No statistically significant difference
Sheng J et al., 2022 [39]	UniDental (haptic VR)	2-arm RCT	38 Year-3 dental students	Inlay tooth preparation accuracy (virtual VR simulation vs jaw model training)	Student attitudes toward learning modality	Favours hybrid arm
Singh V et al., 2024 [40]	Immersive VR simulation	2-arm RCT	60 Year-3 dental students	Overall clinical skill performance score (immersive VR training vs conventional)	Student confidence	Favours AR/VR
Tubelo RA et al., 2016 [41]	Virtual learning object	Cluster RCT	46 dental students	Zinc phosphate cement (ZPC) theoretical knowledge and manipulation skill score (virtual learning object vs traditional)	Not reported	Favours AR/VR
Vannaprathip N et al., 2020 [42]	SDMentor (haptic VR)	2-arm RCT	36 Year-5 dental students	Accuracy of endodontic access decision-making (SDMentor VR intelligent tutor vs control)	Cognitive workload, situational awareness	Favours AR/VR
Vincent M et al., 2020 [43]	Virteasy (haptic VR)	2-arm RCT	88 Year-1 dental students	Class I cavity preparation accuracy (Virteasy haptic VR vs phantom training)	Not reported	No statistically significant difference
Yari A et al., 2023	AR book	2-arm RCT	40 dental students	Inferior alveolar nerve block skill score and learner concentration (AR book vs traditional text)	Learner satisfaction	Favours AR/VR
Zhan Y et al., 2022	Dynamic navigation (AR)	Pilot RCT	6 senior dental students	Implant placement accuracy (augmented-reality dynamic navigation vs freehand)	Self-confidence	Favours AR/VR
Zhang BP et al., 2020 [44]	UniDental (haptic VR)	Multi arm RCT	80 OMFS students	Implant placement accuracy and operative performance (haptic VR simulation vs hybrid training)	Theoretical knowledge test	Favours hybrid arm
Zhang J et al., 2021 [45]	Virtual simulation platform (periodontology)	4-arm RCT	60 dental students	Periodontal instrument debridement skill and knowledge score (periodontology VR simulation vs jaw model)	Student feedback and perceived learning value	Favours hybrid arm

Abbreviations: Augmented Reality (AR); Virtual Reality (VR); Randomized Controlled Trial (RCT); Oral and Maxillofacial Surgery (OMFS)

**Table 3:** Taxonomy of Haptic simulator devices

Tool	Article	AR/VR	Specialization	Objective	Region of Interest	Availibility	Setup	Visual Quality	Haptic Feedback	Tracking Accuracy	Anatomical Realism	Ease of Use	Performance Analysis	Scenario Diversity
NO NAME	Dwisaptarini et al., 2018 [46]	VR	OMFS	Training and performing visual surgical planning in OMFS			VR operates on a 2.8-GHz Pentium 4 PC, with 256 MB RAM; 2 Omni haptic devices	13-inch computer monitor		Not reported	High – Based on 3D micro-CTs of 10 extracted permanent molars with deep carious lesions	Not reported	Participants were assessed by experts and scored	Not reported
UniDental VS System	Fu et al., 2024 [30]	VR	OMFS	Improve teaching ultrasonic scaling technique			Main tower with tools and functional interface	3D mixed reality display system, through the observation window or by wearing headsets		Not reported	High – Based on 3D scan with smartphone and intraoral scan to make custom or patient specific training cases	Moderate – Introductory guidance needed	Compared to traditional group and quail egg; significantly higher	Custom training cases and patient specific cases
Unidental V1.0	Li et al., 2025 [33]	VR	Dentistry	Dental veneer tooth preparation			Not reported	2D display		High – Soft tissue force feedback	Moderate – Lacks gingival dynamics, salivary flow, tongue mobility, etc	Not directly stated – Mentions technical issues and need for recalibration	Evaluated using a 100-point rubric; includes marginal integrity, depth, contour, smoothness	Allows practice on different tooth positions and complex cases
Simodont Dental Trainer	Li et al., 2025; de Boer et al., 2017; Hamama et al., 2024; Murbay et al., 2020 [29, 31, 33, 36]	VR	Dentistry	Dental veneer tooth preparation; Effect of force feedback (FFB) on performance and satisfaction; Detect and treat caries; Operative dentistry procedures			Haptic handpiece, 3D display, virtual drill, simulated tooth block	Includes 3D vision and adjustable zoom		Moderate – Precise tracking of drill position and pressure, better force feedback for tooth preparation; lacks soft tissue feedback	High – Provides realistic visualization and tactile experience; includes tissue-specific hardness and visual cues; lacks gingival dynamics, salivary flow, tongue mobility, etc	High – Students preferred working with FFB; some fatigue noted; occasional technical issues and need for recalibration	Detailed analysis using a 100-point rubric; Collected metrics on student performance; Self-assessment, detailed digital analysis of cavity prep (depth, width, centering, smoothness)	Allows practice on different tooth positions and complex cases, different difficulty levels available
SIMtoCARE Dente	Philip et al., 2023; Daud et al., 2023 [28, 37]	VR	Dentistry	Pulpotomy procedures; Cavity preparation and caries removal			Physical handpiece (stylus) and dental mirror with virtual display on monitor	Not reported		Not reported	High – Visual realism rated high by 72% of students; based on scanned extracted teeth, with caries texture simulation; tactile sensations moderate (difficult to distinguish enamel/dentine)	Moderate – Students noted differences in grip, weight, and manoeuverability vs. real tools; lack of finger rests and lag when navigating images	Tracks procedural steps, quality scores, time taken and instructor prompts; time spent, percentage of structure removed, deviation from task	Multiple exercises and procedures with difficult complexity; focused on Class I cavity preparation and caries removal
hapTEL	San Diego et al., 2022 [38]	VR	Dentistry	Dental cavity preparation and caries removal			3D glasses, haptic device with handpiece and mirror	Not reported		Moderate – Tracks handpiece angulation and movement)	Moderate – 3D virtual teeth with layered structure	Low – Perceived as harder to use than phantom head	Scoring rubric, tutor observation, statistical analysis	Different tasks with varying difficulty and tooth positions
UniDental-MU01	Sheng et al., 2022 [39]	VR	Dentistry	Prosthodontics			Computer based system	Not reported		Not reported	High – Includes extracted teeth and scanned models for evaluation	Not reported	Includes theoretical and practical scores, scanned tooth forms, and Likert-scale feedback	Focused on inlay preparation on premolars
SDMentor	Vannaprathip et al., 2025 [42]	VR	Dentistry	Surgical decision making in dentistry (root canal treatment)			HTC Vive Pro Eye headset, Geo-Magic Touch haptic devices	2880 × 1600 resolution, stereoscopic 3D		High – Uses collision detection and symbolic action recognition	High – Based on micro-CT scans of real teeth	Not directly stated – Requires VR headset and haptic devices	Logs actions, evaluates decisions, tracks learning progress	Supports multiple intraoperative stages; customizable scenarios
Virteasy	Vincent et al., 2020 [43]	VR	Dentistry (restorative)	Cavity preparation			Windows 7 PC, touchscreen, 3D stereoscopic glasses, Geomagic Touch X haptic device, foot pedal	3D stereoscopic display (Estar America ESG6100 glasses)		High – Simulator records % inside/outside target, drilling time, etc	High – Includes realistic tooth models and cavity shapes (e.g., Black’s Class II)	Not directly stated – Students received demonstrations and briefings	Objective metrics (e.g., % inside/outside, time), subjective scoring by instructors	Focused on Class II cavity with mesial and prophylactic extensions

Images of tooth, bone, soft tissue, academic, commercial, yes and no (freepik.com and Lucide.dev, November 2025)

TeethBoneSoft tissueAcademicCommercialYesNo

Abbreviations: Augmented Reality (AR); Virtual Reality (VR); Oral and Maxillofacial Surgery (OMFS); Force Feedback (FFB); Cone Beam Computed Tomography (CBCT)

**Table 4:** Taxonomy of Head-mounted devices

Tool	Article	AR/VR	Specialization	Objective	Region of Interest	Availibility	Setup	Visual Quality	Haptic Feedback	Tracking Accuracy	Anatomical Realism	Ease of Use	Performance Analysis	Scenario Diversity
3DOVR-DR	Im et al., 2025 [47]	VR	Dental Radiography	Dental radiography procedures			Oculus Quest 2 with controller	Not reported		Not reported	High – Realistic visual modelling	High – Commercially available device	Errors indicated with visual cues	Includes different teeth & different procedural steps
NO NAME	Mansoory et al., 2022 [34]	VR	Dentistry	Prosthodontics – neutral zone and teeth arrangement			Gear VR headset (Samsung), smartphone, Unity-based app, 3D cube navigation	Uses 3D cube with multi-angle video		Not reported	Moderate – Based on multi-angle video capture of real procedures	Mixed – Some confusion and incompatibility with glasses noted	Pre-/posttests and practical skill checklists	Focused on neutral zone and teeth arrangement
NO NAME	Peters et al., 2023 [48]	VR	Dentistry & Medicine	Surgical suturing			Oculus Quest 2	Not reported		Not reported	Low – Low-fidelity model used	Mixed – Generally positive; some discomfort noted	Optical flow, number of sutures	Not reported

Images of tooth, bone, soft tissue, academic, commercial, yes and no (freepik.com and Lucide.dev, November 2025)

Teeth Bone Soft tissueAcademic Commercial Yes No

Abbreviations: Augmented Reality (AR); Virtual Reality (VR); Oral and Maxillofacial Surgery (OMFS); Force Feedback (FFB); Cone Beam Computed Tomography (CBCT)

**Table 5:** Taxonomy of software

Tool	Article	AR/VR	Specialization	Objective	Region of Interest	Availibility	Setup	Visual Quality	Haptic Feedback	Tracking Accuracy	Anatomical Realism	Ease of Use	Performance Analysis	Scenario Diversity
REPs-VS platform	Hu et al. 2025 [32]	VR	Dentistry	Teach regenerative endodontic procedures			Web-based platform, PC/mobile	Not reported		Not reported	Moderate – Good visual and operational realism but lacks tactile precision	High – Software is accessible from multiple devices; no physical device or weight concerns	Study hours, progress, and mastery	Virtual patient with complex scenarios and gamification elements
Zhonghui	Huang et al. 2025 [49]	VR	Dentistry	Operative dentistry procedures	Not reported		Desktop-based VR (not headset)	Not reported		Not reported	Not reported	Not reported	Automated scoring, eye-tracking, questionnaires	Not reported
NO NAME	Lu et al., 2022 [50]	VR	Dentistry (pediatric)	Pulpotomy			Computer-based; no headset or physical device mentioned	Not reported		Not reported	Moderate – Simulates clinical environment and procedures; lacks full anatomical diversity	High – accessible anytime, repeatable, interactive	Includes learning tracking, scoring, and retrospective analysis	Focused on specific clinical scenarios (pulpotomy)
Dental Simulator	Mladenovic et al., 2019 [35]	AR	Dentistry	Improve Inferior Alveolar Nerve Block (IANB) learning			Mobile device + AR mode + VR goggles + plastic jaw model	Not reported		Not reported	Moderate – AR overlays on plastic jaw model; improved visualization of reference points	High – positive student feedback	Questionnaire + time measurement + success rate	Single scenario: Inferior Alveolar Nerve Block
NO NAME	Singhet al., 2024	VR	Dentistry	Clinical skills and confidence			Desktop-based VR	Not reported		Not reported	Not reported	Not reported	Standardized checklist, real-time feedback, confidence questionnaire	Multiple clinical scenarios available
Virtual Learning Object (VLO)	Tubelo et al., 2016 [41]	VR	Dentistry	Dental materials (Zinc Phosphate Cement) manipulation			Laptop or desktop computer	Not reported		Not reported	Low – Focus on material manipulation, not anatomy	High – Survey showed high acceptance (84.6% rated “very frequently”)	Automatic scoring in simulation; pre/post theoretical tests + lab tests (film thickness, setting time)	Single scenario: Zinc Phosphate Cement manipulation
Zappar	Yari et al., 2024 [51]	AR	Dentistry	Local anaesthesia			Smartphone or tablet (no headset)	Not reported		Not reported	High – Based on Sobotta Atlas, 3D models of head anatomy	Moderate – Smartphone-based, no mention of weight/ergonomics	Written exam, practical score, injection time	Not reported
Dynamic navigation system	Zhan et al., 2021 [52]	VR	Dentistry	Dental implant placement (dynamic navigation vs. traditional freehand)			Computer monitor, stereoscopic camera, handpiece with tags	Real-time video feedback; no specific resolution, refresh rate, or field of view stated		High – Real-time tracking of drill and jaw position; deviation measured in 3D	Moderate – Simulated model with radiographic markers; not full soft tissue simulation	High – Students rated ease of use highly (mean score 9.1); no mention of weight or ergonomics	3D deviation at implant platform/apex and axis; statistical analysis of improvement	Single implant site (maxillary left first molar); no mention of varied cases
UniDental-MS01	Zhang et al., 2020 [44]	VR	OMFS	Preclinical implant training			Computer based system	Not reported		Not reported	Moderate – Simulates implant site and procedure	Moderate – Students found it engaging but not superior aline	Theoretical scores, implant accuracy	Mandibular molar implant only
UniDental	Zhang et al., 2021 [45]	VR	Dentistry	Periodontology—supragingival scaling and periodontal examination			VR system (UniDental), possibly with headset and controllers	Not reported		Not reported	Moderate – Includes periodontal structures and procedures	Moderate – Students reported high satisfaction	Theoretical exams, operation scores, plaque index, satisfaction survey	Includes periodontal probing, supragingival scaling, oral hygiene instruction

Images of tooth, bone, soft tissue, academic, commercial, yes and no (freepik.com and Lucide.dev, November 2025)

Teeth Bone Soft tissueAcademic Commercial Yes No

Abbreviations: Augmented Reality (AR); Virtual Reality (VR); Oral and Maxillofacial Surgery (OMFS); Force Feedback (FFB); Cone Beam Computed Tomography (CBCT)

## Risk of bias detection

Two reviewers independently assessed the risk of bias and methodological quality. For RCTs, the Cochrane RoB 2 tool [53] was used. Disagreements were resolved by consensus. The results were summarized in tables and considered in the interpretation of the evidence.

## Results

### Study selection

The systematic search initially identified 390 records, from which 29 duplicates were removed, yielding 361 unique records for screening. After title and abstract screening, 30 full-text articles were assessed for eligibility, of which 25 randomized controlled trials met all inclusion criteria and were included in this review. The study selection process is detailed in the PRISMA flow diagram (Fig. 1). The included studies are summarized in Table 2. Most trials were conducted in undergraduate dental education, whereas a smaller subset focused on OMFS training (22 studies in general dentistry vs. 3 in OMFS).

### Risk of bias for included studies

**Fig. 2:**
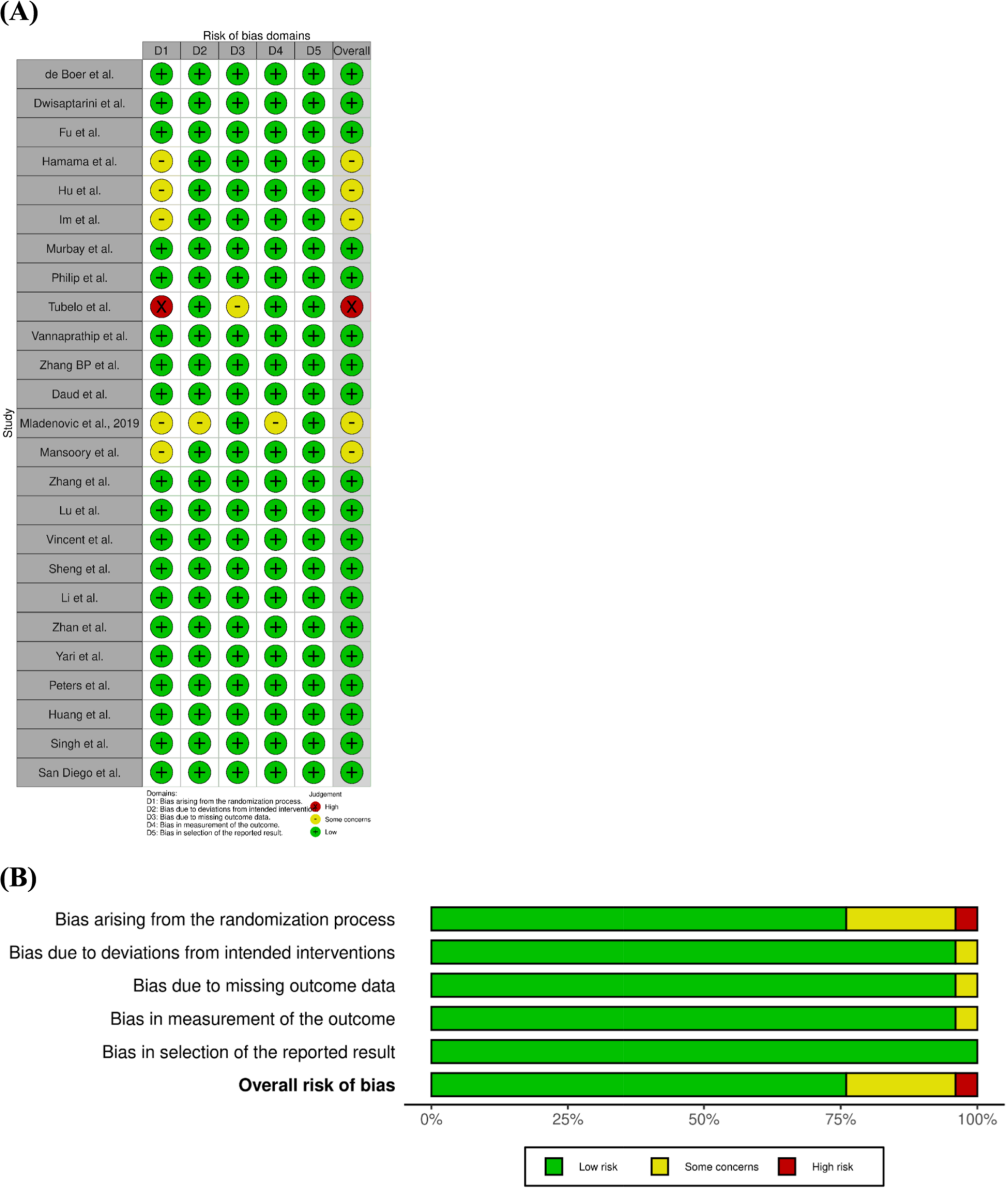
Risk of bias with the ROB-2 tool. **A** Traffic light plot; **B** summary plot

A risk-of-bias assessment was performed using the Cochrane RoB-2 tool. The overall methodological quality of the included randomized controlled trials was generally favorable. Nineteen of the included studies (76%) were judged to be at low risk of bias. Five studies (20%) were assessed as having some concerns [31, 32, 34, 35, 47]. One study (4%) was judged to be at high risk of bias [41]. The most frequent concerns were related to the randomization process (RoB-2 Domain 1). In contrast, deviations from intended interventions were generally judged to be at low risk of bias. Outcome measurement was also assessed as low risk across most studies. Selective reporting did not emerge as a major source of bias. Despite these limitations, the overall body of evidence can be considered methodologically sound. Nevertheless, the findings should be interpreted with appropriate caution (see Fig. 2).

### Characteristics of the included studies

For each included RCT, the following data were extracted:

- Study Characteristics: Publications ranged from 2015 to 2025 (with most after 2020) and were conducted across Europe, Asia, and North America. Geographically, roughly half of the studies were from Asian institutions (e.g., China, South Korea, India, Iran, Thailand), about one-third from Europe (e.g., the UK, Netherlands, France), and the remainder from North America (United States) with one from South America (Brazil). Learner populations were predominantly novice dental trainees, mainly undergraduate dental students (*n* = 22 studies, 88%) and a smaller number of OMFS residents or surgeons in training (*n* = 3, 12%).

- AR/VR Tools: The interventions fell into three broad categories: (a) Haptic simulators (*n* = 13), such as Simodont, UniDental, SIMtoCARE Dente, and Virteasy, which are high-fidelity VR systems providing force-feedback via haptic devices; (b) Head-mounted displays (HMD) (*n* = 3), including immersive VR headsets like Oculus Rift and mixed-reality AR glasses like Microsoft HoloLens, offering an interactive 3D visual environment (often without tactile feedback); and (c) Software-based platforms (*n* = 9), such as mobile AR applications (e.g., AR textbooks), web-based VR modules, or computer-guided navigation systems. These software-based tools typically run on standard computers, tablets, or smartphones without specialized hardware. In terms of reality modality, the vast majority of included studies evaluated VR-based training tools, whereas a minority investigated augmented or mixed-reality approaches. Notably, only two studies (8%) employed AR systems (e.g., an AR anatomy textbook and an AR dynamic navigation system for implant placement), and one study (~4%) utilized a mixed-reality headset (HoloLens), while the remaining ~88% of trials focused on purely VR simulations.

- Reference standards: The control or comparison groups received traditional training modalities. These included conventional phantom head simulators with plastic teeth, instructor-led didactic teaching or videos, cadaver or model workshops, and other non-AR/VR simulation methods.

- Performance metrics: Outcome measures encompassed a range of objective technical performance endpoints and subjective learning outcomes. Common primary outcomes were preparation quality scores (e.g. cavity or crown prep grading rubrics), amount of residual errors (such as remaining caries or calculus), procedure completion time, diagnostic or treatment planning accuracy, and for implantology studies, the positional/angular deviation of implant placement. Several studies also assessed secondary outcomes like written exam scores (knowledge retention), learner self-confidence, satisfaction, or other perception questionnaires, to evaluate the educational impact of AR/VR beyond technical skill performance. Detailed characteristics and outcomes for each study are provided in Tables 3, 4 and 5.

### Findings of the included studies

Across the included randomized controlled trials, AR/VR interventions were evaluated using three primary technological approaches: haptic simulators, HMDs, and software-based platforms (Tables 2, 3, 4 and 5). Simulation fidelity varied substantially across studies and refers to the extent to which a system reproduces real-world task demands through visual realism, tactile feedback, and procedural interactivity. Because both fidelity and educational design differed markedly between trials, results are reported descriptively by technology type rather than pooled quantitatively.

Haptic simulators were predominantly used to train psychomotor dental skills such as cavity preparation, caries removal, periodontal scaling, veneer or inlay preparation, and restorative procedures. Objective performance outcomes—most commonly preparation accuracy, residual error, or completeness of removal—were superior to conventional training in the majority of trials (Table 2) [28–31, 33, 36–39, 42, 43, 46]. Studies incorporating force-feedback and structured, real-time guidance more frequently reported improvements in accuracy and learning efficiency [29, 30, 42], whereas several trials observed no statistically significant difference between haptic VR and traditional phantom-head training [33, 37, 38, 43, 46]. Differences across trials coincided with variation in the level of guidance, interactivity, and training exposure implemented. Procedural time was inconsistently reported and did not demonstrate a uniform advantage for either approach. Learner confidence and perceived usefulness were more frequently higher in the AR/VR arms. One study suggested that a hybrid sequence combining VR with conventional practice yielded the most favorable outcomes [39].

Head-mounted display–based interventions primarily targeted spatial understanding, anatomy learning, and selected technical skills such as suturing or crown preparation. These trials most often demonstrated improvements in anatomy recognition, spatial orientation, or task execution compared with conventional instruction, alongside consistent gains in learner confidence [34, 47, 48]. In contrast, one HMD study reported no statistically significant difference in objective performance outcomes relative to controls [48]. These neutral findings likely reflect the absence of haptic feedback and the short duration of exposure rather than inferiority of immersive visualization itself. Effects on procedural time were variable and not consistently assessed across studies.

Software-based platforms, including desktop VR environments, web-based simulators, and AR learning objects, were mainly applied to knowledge acquisition, diagnostic reasoning, procedural planning, and selected clinical skills. These interventions generally improved examination scores, diagnostic accuracy, or decision-making performance compared with conventional teaching methods [32, 35, 40, 44, 45, 49–52]. However, effects on hands-on technical accuracy were less consistent than for haptic systems, and a small number of trials reported outcomes comparable to traditional models [45]. This variability reflects substantial differences in software complexity, interactivity, and educational intent, ranging from low-interaction AR overlays to platforms incorporating tracking and structured analytics.

Across all three technology categories, standardized reporting of outcome measures was not feasible. Although outcomes could conceptually be grouped into accuracy, procedural time, learner confidence, and examination or knowledge scores, the specific metrics, scoring systems, tasks, and assessment instruments varied widely between studies. Consequently, results could not be meaningfully harmonized across trials, and synthesis was limited to direction-of-effect reporting within each modality. Despite this heterogeneity, AR/VR interventions were consistently at least as effective as conventional training approaches, with the most robust and reproducible gains observed in objective performance accuracy and learner confidence (Tables 2, 3, 4 and 5).

## Discussion

### Summary of evidence

The integration of AR and VR technologies in surgical training, particularly within dentistry and OMFS, highlights promising advancements in skill acquisition, learner satisfaction, and innovative educational methods.

As highlighted by Serrano et al., the immersive nature of VR, particularly through advanced simulation tools such as the Simodont Dental Trainer, significantly contributes to refining theoretical knowledge and technical skills among trainees in a safe environment [47]. This aligns with broader findings by Dalanon et al., who reported that the use of AR and VR technologies enhances procedural familiarity, resulting in improved learner engagement and satisfaction as these modalities allow for risk-free practice [54]. The review of the current literature supports the argument that AR/VR contributes positively to the educational experiences of surgical trainees. Trainers and trainees frequently value the ability for instant feedback and practice in risk-free environments. The potential of these tools to increase motivation, enthusiasm, and self-confidence also emphasizes their value as educational tools [55, 56].

### Clinical translation

An essential aspect of evaluating AR and VR training lies in the transferability of acquired skills to real clinical environments. Evidence indicates that competencies acquired through immersive simulation, such as hand–eye coordination, spatial awareness, and decision-making, translate effectively to improved performance, accuracy, and confidence in the operating room [15, 20]. Haptic VR simulators in particular strengthen psychomotor control and procedural fluidity, enabling learners to reproduce clinically relevant manoeuvres with greater consistency [8, 15]. Within OMFS, AR systems that support intraoperative guidance and preoperative planning exemplify how virtual training environments can bridge the gap between simulation and real surgery [8, 26]. These tools facilitate continuous rehearsal and reflection, encouraging a shift from isolated technical skill acquisition towards integrated procedural understanding. This holistic approach positions AR and VR not only as adjuncts for training but also as components of a digital surgical ecosystem. It allows for planning, connection of diagnostics, execution, and postoperative evaluation, therefore fostering competency-based learning and improved patient centred care [26, 57, 58].

Beyond indirect impact, virtual reality technology can have benefits for patients in a direct manner as well. Different VR tools suggest to be effective in reducing anxiety before dental treatment [59]. Although studied in verious patient populations, the application of VR as a distractive tool has been mainly used in pediatric patients. It has been proven to alleviate anxiety and pain, resulting in a more positive attitude towards dental procedures. This finally results in overall shorter procedures and improved patient satisfaction [60].

Notably, orthognathic and oncologic surgeries in OMFS have begun to adopt AR and VR for enhanced planning and intraoperative guidance. On the one hand, AR-based navigation in orthographic surgery can overlay virtual osteotomy lines to streamline execution [61]. In head and neck oncology, however, recent studies have demonstrated that AR guidance can significantly increase the precision of tumor resection by clearly delineating surgical margins in real time [21]. Although these applications are still emerging in OMFS, implementations of this type of surgery have achieved success in other surgical fields. In urologic surgery, for example, AR overlays combined with AI-driven planning have already been used to revolutionize procedure planning and intraoperative accuracy. This finding indicates a promising future where similar AI-supported AR navigation could enhance safety and precision [62, 63]. In implant dentistry, computer-assisted guidance, particularly dynamic navigation, consistently improves placement accuracy and attenuates the novice–expert performance gap [11, 64]. Building on this, AI-enabled “virtual-patient” generation from cone beam CT (CBCT)/intraoral scanner (IOS) (automated segmentation with prosthetically driven planning) can be exported one-to-one to dynamic navigation for real-time, plan-concordant execution, enabling a plan–simulate–navigate training loop [65, 66].

### Barriers and implementation factors

The implementation possibilities of these AR and VR tools are highly variable, ranging from software to head-mounted devices with haptic feedback. On the one hand, high variability causes heterogeneity in study results and complicates comparisons across different platforms, making it difficult to choose the right tools from such a broad range. On the other hand, their distinct adaptivity allows universities and hospitals to invest in tools that fit their needs and curriculum, resulting in more profitable implementation [46].

Despite multiple implementation possibilities, there is a lack of generalized application due to multiple adoption barriers. Foremost, few data concerning cost-effectiveness are available. Although favourable cost-analyses in other specialties have been published, a high initial cost is a remaining implementation barrier [67]. Second, there are logistical challenges, including the need for dedicated rooms, specialized hardware, and technical support. However, a recent review on the adoption of haptic-enhanced VR training reported that 66% of the schools that bought a VR simulator would recommend this training to other schools [68].

### Limitations

Notwithstanding the encouraging advancements in AR/VR applications, methodological heterogeneity and the prevalence of small sample sizes in existing studies limit the generalizability of these findings.

### Future directions

Future research on this topic should include long-term RCTs with adequate sample sizes, training protocols, standardized outcome measures, and cost-effectiveness analyses. Moreover, these studies should focus not only on skill acquisition but also on user satisfaction.

## Conclusion

This systematic review shows that augmented reality and virtual reality provide training outcomes that are noninferior and often superior to conventional methods in dentistry and oral and maxillofacial surgery. Across 25 randomized trials, these technologies improved objective procedural performance, spatial understanding, and learner confidence. However, the evidence is limited by heterogeneous study designs, small sample sizes, and a lack of data on long-term skill retention and cost-effectiveness. Although augmented and virtual reality are increasingly integrated into educational curricula and allow safe, flexible skill acquisition, their full educational value will depend on standardized outcome measures, adequately powered multicenter studies, and robust economic evaluations.

## Supplementary Information

Supplementary Material 1

## Data Availability

All the data generated and analysed during this review are included in this published article. The extraction sheets and screening decisions are available upon request from the corresponding author.

## Abbreviations

AI: Artificial intelligence
AR: Augmented reality
CBCT: Cone-beam computed tomography
DOI: Digital object identifier
HMD: Head Mounted Display
IOS: Intraoral scanner
OMFS: Oral and maxillofacial surgery
OSF: Open Science Framework
RCT: Randomized controlled trial
VR: Virtual reality
XR: Extended reality
